# AT-NeuroEAE: A Joint Extraction Model of Events With Attributes for Research Sharing-Oriented Neuroimaging Provenance Construction

**DOI:** 10.3389/fnins.2021.739535

**Published:** 2022-03-07

**Authors:** Shaofu Lin, Zhe Xu, Ying Sheng, Lihong Chen, Jianhui Chen

**Affiliations:** ^1^Faculty of Information Technology, Beijing University of Technology, Beijing, China; ^2^Beijing Institute of Smart City, Beijing University of Technology, Beijing, China; ^3^Engineering Research Center of Digital Community, Beijing University of Technology, Beijing, China; ^4^Beijing Key Laboratory of Magnetic Resonance Imaging (MRI) and Brain Informatics, Beijing University of Technology, Beijing, China; ^5^Beijing International Collaboration Base on Brain Informatics and Wisdom Services, Beijing University of Technology, Beijing, China

**Keywords:** neuroimaging provenance, neuroimaging text mining, event extraction, attribute extraction, deep adversarial learning

## Abstract

Provenances are a research focus of neuroimaging resources sharing. An amount of work has been done to construct high-quality neuroimaging provenances in a standardized and convenient way. However, besides existing processed-based provenance extraction methods, open research sharing in computational neuroscience still needs one way to extract provenance information from rapidly growing published resources. This paper proposes a literature mining-based approach for research sharing-oriented neuroimaging provenance construction. A group of neuroimaging event-containing attributes are defined to model the whole process of neuroimaging researches, and a joint extraction model based on deep adversarial learning, called AT-NeuroEAE, is proposed to realize the event extraction in a few-shot learning scenario. Finally, a group of experiments were performed on the real data set from the journal PLOS ONE. Experimental results show that the proposed method provides a practical approach to quickly collect research information for neuroimaging provenance construction oriented to open research sharing.

## Introduction

Open and FAIR (Findable, Accessible, Interoperable, and Reusable) sharing (Poldrack and Gorgolewski, 2015; Abrams et al., 2021) of neuroimaging data has been widely recognized and has been a concern in computational neuroscience. From fMRIDC (Van Horn et al., 2001) and OpenfMRI (Poldrack and Gorgolewski, 2015) to OpenNeuro (Gorgolewski et al., 2017), a series of public neuroimaging databases have been constructed and have played an important role in brain and intelligence researches. Only taking the INDI database as an example, by March 22, 2017, Google Scholar search results showed that 913 publications clearly indicated the use of INDI data, with a total of 20,697 citations (Milham et al., 2018). Besides sharing raw experimental data, research information sharing^1^ has also become an important way of neuroimaging data sharing (Poline et al., 2012), which can effectively avoid various challenges of sharing raw experimental data, such as cost, privacy protection, and data ownership. BrainMap^2^ collects the published information related to brain activated areas from the neuroimaging article to support coordinate based meta-analysis (Laird et al., 2005). Neurosynth^3^ provides a specialized tool to automatically extract coordinates of activated brain area and research topic information from published neuroimaging articles and shared them by a unified platform (Yarkoni et al., 2011). NeuroVault^4^ collected non-threshold statistical graphs and related information from different computational neuroscience to support graph based meta-analysis (Gorgolewski et al., 2016). These efforts on sharing neuroimaging research information enrich the content and method of neuroimaging data sharing and effectively support the brain and intelligence studies in computational neuroscience. For example, statistics show that the number of papers based on the neurosynth platform has reached 14,371 by July 2018. Related studies are involved with the brain mechanism of cognitive states (Alcalá-López et al., 2017), functional analysis of brain areas (Genon et al., 2018), large-scale brain structure-function mappings (Bolt et al., 2020), and so on.

Provenances (Moreau et al., 2008) are a research issue of neuroimaging data sharing Poldrack and Gorgolewski (2014) pointed out that sharing task-based fMRI (functional magnetic resonance imaging) data requires the appropriate descriptions of how they were acquired, including not only the MRI acquisition parameters but also the specific order and timing of stimulus presentation during the task, and how they have been transformed. These descriptions are provenances. The special topic “Collaborative efforts for understanding the human brain” of the journal *Frontiers in Neuroinformatics* emphasized that the key to controlling the re-executability of the publication of computational neuroscience is the generation and reporting, at all stages of the process, machine readable provenance documentation (Kennedy et al., 2019). From the data model (Keator et al., 2013; Maumet et al., 2016), the management tool (Keator et al., 2019) to the acquisition platform (Gorgolewski et al., 2016), an amount of work has been done to construct high-quality neuroimaging provenances in a standardized and convenient way. The neuroimaging provenances have been not only metadata of task-based experimental data but also structured descriptions of research cases in computational neuroscience, which collect massive research information about the whole research process of computational science to support multi-aspect resource integration, fast hypothesis generation, large-scale metadata analysis, strict result evaluation, etc., for realizing open and FAIR neuroscience (Abrams et al., 2021). However, existing studies on neuroimaging construction mainly adopt experts or process recording-based methods to obtain neuroimaging provenances. All methods are centered on the actual experimental data. Besides them, open research sharing in computational neuroscience still needs one new way to automatically and quickly extract provenance information from rapidly growing published sources.

Neuroimaging text mining recognizes important information from neuroimaging texts, especially neuroimaging articles, and provides a practical way to automatically extract provenance information from published sources. In recent years, related studies mainly focused on recognizing neuroimaging entities (Abacha et al., 2017; Shardlow et al., 2018; Riedel et al., 2019), hierarchical terminology system (Huangfu et al., 2020), and span of interests (Zhu et al., 2020). Because extracted information is mainly some words or phrases, and lacks rich relations, especially non-taxonomical relations, these studies can only obtain some fragmented key points of the research process and cannot provide a vivid process description. Hence, it is necessary to develop a new technology of neuroimaging text mining for research sharing-oriented neuroimaging provenance construction.

Based on this observation, this paper proposes a literature mining-based approach for neuroimaging provenance construction. The rest of this paper is organized as follows. Section “Related Work” summarizes previous work-related to neuroimaging provenances, neuroimaging text mining, and biomedical event extraction. Section “Contributions” introduces our contributions. Section “Materials and Methods” describes the proposed method. Experiments and results are presented in section “Results” to validate the effectiveness of the proposed method. Finally, section “Discussion” gives concluding remarks.

## Related Work

### Neuroimaging Provenances

Provenances can be described in various terms depending on the domains where they are applied (Simmhan et al., 2005). Buneman et al. (2004) defined provenances as the description of the origins of data and the process by which it arrived at the database. Lanter (1991) characterized provenances as information describing materials and transformations applied to derive the data. Greenwood et al. (2003) viewed provenances as metadata recording the process of experiment workflows, annotations, and notes. Simmhan et al. (2005) defined provenances as information that helped determine the derivation history of a data product. Related studies have been widely concerned by many domains. Science magazine pointed out that provenance is an important element of service quality control (Foster, 2005). The NSF (National Science Foundation, Alexandria, VA, United States) Advisory Committee believed that a robust and stable cyberinfrastructure should support provenances (Task Force on Grand Challenges, 2011). The World Wide Web Consortium standard of provenances, called PROV-DM (Provenance Data Model),^5^ has been released in 2013. A series of international conferences about provenances, such as IPAW (International Provenance and Annotation Workshop, since 2006) and TaPP (Workshop on Theory and Practice of Provenance, since 2009), has attracted a large number of researchers.

Computational neuroscience is an important application domain of provenances. Neuroimaging provenances are the research focus. As early as 2006, the first fMRI data provenance challenge has been held (Moreau et al., 2008). A group of minimum provenance information (MI) guidelines (Gibson et al., 2008; Poldrack et al., 2008; Frishkoff et al., 2011) has been proposed for different types of neuroimaging data. Guided by these MI guidelines, the main neuroimaging data model, such as DICOM (Digital Imaging and Communications in Medicine)^6^ and XCEDE (XML-based Clinical and Experimental Data Exchange),^7^ provides the support for representing and storing provenance information. However, the provenance information storage embedded into experimental data models hampers the representation of more complex relationships among data (Ruiz-Olazar et al., 2016). Provenances independent from experimental data have drawn attentions. The representative research work is NIDM (Neuroimaging Data Model), which provides a complete description of provenance for computational neuroscience, from raw data to the final results including all the steps in between (Maumet et al., 2016). NIDM builds upon PROV-DM and extends it with terms found in XCEDE and DICOM headers. It consists of NIDM-Experiment, NIDM-Workflow, and NIDM-Results; NIDM-Experiment and NIDM-Workflow (Keator et al., 2013) model acquisition and processing of experimental data, respectively. NIDM-Results (Maumet et al., 2016) focuses on neuroimaging statistical results along with key image data summarizing the experiment.

In recent years, many studies are working to support the construction of neuroimaging provenances based on NIDM for realizing machine readable descriptions of the data collection, the processing workflow and environment, and the statistical procedures and results. The export and import of NIDM documents have been realized in many widely used neuroimaging tools and platforms, such as SPM, FSL, and NeuroVault. Keator et al. (2019) developed a python-based API (PyNIDM) which followed the simple organizational structure of NIDM-Experiment with functions to create, query, export, import, and transform NIDM-Experiment documents. Kennedy et al. (2019) developed the function to generate NIDM based provenances on ReproNim, which is a center for reproducible neuroimaging computation. Maumet et al. (2019) built a JSON-LD representation for NIDM-Results data and exposed it to the neuroimaging data management tool Datalad. With the growth of scale, the applications of neuroimaging provenances also have broken through traditional data descriptions oriented to data sharing (Van Horn et al., 2001) and process recordings oriented to analysis (Dinov et al., 2009), and are moved toward verification of scientific outputs (Arshad et al., 2019), research artifact sharing (Maumet, 2020), etc. Provenance-based research sharing (Yuan et al., 2018), which uses provenances as “research objects” (Belhajjame et al., 2015; Miksa and Rauber, 2017; That et al., 2017) (i.e., digital research artifacts for knowledge sharing and reproducibility in computational experiments), is becoming a trend for open and FAIR neuroscience.

However, provenance-based research sharing needs large-scale and high-quality neuroimaging provenances. Existing studies of neuroimaging provenances mainly adopt experts or process recordings-based construction methods. Public neuroimaging data or derived data sharing platforms, such as BrainMap and NeuroVault, rely on experts’ manual annotations to collect neuroimaging provenance information. The obtained provenances vary dramatically in quality and grow slowly. Even if Brainspell^8^ adopts the crowdsourcing-based annotation mode (Badhwar et al., 2016), the situation has not been fundamentally improved. Aiming at this problem, many neuroimaging tools, such as SPM, FSL, and PyNIDM, have provided the supporting for process-based provenance extraction. They can collect provenance information at the time of data acquisition or processing and do not require any extra effort. Process-based provenance extraction has become the most important means to construct neuroimaging provenances for large-scale data sharing. However, this kind of provenance extraction method is centered on actual experimental data. At present, neuroimaging data sharing still faces some important obstacles, such as privacy and data ownership. Public data account for only a small part of the total data. Similarly, many researchers do not actively generate and share neuroimaging provenances during the data acquisition or processing. Therefore, besides collecting provenance information during data acquisition or processing, it is also necessary to develop one new way to extract provenance information from published sources, especially published scientific articles.

### Neuroimaging Text Mining

Neuroimaging technologies can non-invasively detect the connection between cognitive states and patterns of brain activity, and have received widespread attentions in computational neuroscience. Neuroimaging texts, especially scientific articles, are growing fast. Taking only fMRI (Wegrzyn et al., 2018) as an example, 307 relevant articles have been published in the journal PLOS ONE in 2020. These neuroimaging texts are valuable knowledge resources for studying human intelligence, pathological mechanism of brain and mental diseases, brain-computer interface, and so on. In the last decade, various neuroimaging meta-analysis (Yarkoni et al., 2011; Neumann et al., 2016) and collaborative analysis (Lei et al., 2020) have made great achievements. The value of neuroimaging texts as knowledge resources is already obvious. How to automatically and continuously extract knowledge from neuroimaging texts has become a key issue for open and FAIR neuroscience.

Neuroimaging text mining is an important branch of biomedical text mining and provides an effective approach to extract knowledge from neuroimaging texts. Early studies mainly focused on extracting neuroimaging knowledge for decoding a wide range of cognitive states Naud and Usui (2008) constructed the neuroscience terminology space by using the Vector Space Model and performed k-means based cluster on the abstract of neuroscience articles for extracting cognitive state-related topics. Neurosynth (Yarkoni et al., 2011) recognized terms based on word frequency and used the Naive Bayesian Classifier to predict the occurrence of specific terms based on the whole-brain activation patterns. Poldrack et al. (2013) adopted the Latent Dirichlet Allocation (LDA) method to identify topics of articles from the Neurosynth database and mapped these topics to brain activation data for discovering mechanisms of cognitive states. French et al. (2012) developed a co-occurrence-based method to extract brain regions and their relations from neuroscience articles. Alhazmi et al. (2018) extracted topic words based on word frequency and constructed relations between semantic spaces of topics and brain-activated regions by using correspondence analysis and hierarchical clustering. In order to decode cognitive states, these studies only extracted cognitive states and activated brain regions and their relations from neuroimaging articles. Main adopted text mining technologies are the frequency-based or probabilistic model-based topic learning methods and the clustering-based relation recognition methods. The obtained knowledge was a group of topic words and their relations. Because of adopting an open-domain task to extract unknown types of topics and relations, many general words were often included in topics, resulting in low-quality results of knowledge extraction. For example, neurosynth topic words include many general words, such as “using,” “repeat,” “asked,” and domain general words, such as “magnetic resonance,” “brain” (Abacha et al., 2017). Poldrack et al. (2013) had to use concepts in the Cognitive Atlas (Poldrack et al., 2011) to further filter neurosynth topic words. Such knowledge extraction oriented to the decoding of cognitive states cannot effectively characterize the whole research process for sharing “research objects.”

In recent years, studies on neuroimaging text mining began to expend the knowledge extraction perspective from decoding cognitive states to characterize the whole research process. The adopted methods also change from topic modeling to named entity recognition. Ben Abacha et al. (2017) adopted the rule-based method and various conditional random field (CRF)-based methods to recognize fifteen functional neuroimaging entity categories, including gross brain anatomy, functional neuroanatomy, medical problem, stimuli, and responses, etc. Shardlow et al. (2018) recognized various entities, including brain regions, experimental values, neuron types, etc., by using active and deep learning, for curating researches in computational neuroscience. Riedel et al. (2019) completed a comprehensive evaluation on recognizing various entities related to the cognitive experiment, which are defined by the Cognitive Paradigm Ontology (Turner and Laird, 2012) and involved with behavioral domain, paradigm class, instruction, stimulus modality, etc., based on multiple corpus features and various classifiers, including Bernoulli naïve Bayes, k-nearest neighbors, logistic regression, and support vector classifier. Huangfu et al. (2020) extracted the term list of the neuroscientific research process from the PubMed Central database, for creating neuroscientific knowledge organization system (i.e., hierarchical terminology system). Zhu et al. (2020) proposed the hierarchical attentive decoding to extract a span of interests (i.e., terms about neuroscientific research process) from neuroscience articles, to predict research species. Whether it is the entity, method term, or research interest, the extracted knowledge in current studies of neuroimaging text mining is no longer limited to cognitive states and brain regions but extended to the whole research process, from the experiment to analysis. Furthermore, most of these studies transformed the open-domain topic modeling task to the close-domain entity recognition task with predefined entity/term/interest types from external domain knowledge, such as Cognitive Paradigm Ontology. By this kind of transformation of task definition, the shortcoming of general word noises in early studies of neuroimaging text mining can be effectively solved. Therefore, rapid growth neuroimaging articles, especially open access articles, and the maturing technologies of neuroimaging text mining provide a practical approach for continuous and rapid collection of neuroimaging provenance information.

However, for provenance-based research sharing, existing studies on neuroimaging text mining still have the following shortcoming. Words or phrases are the main form of extracted knowledge. Because of lacking the relation structure among words, these words or phrases can only show some fragmentary and key points of the research process and cannot provide a vivid process description. Hence, it is necessary to develop a new technology of neuroimaging text mining for research sharing-oriented neuroimaging provenance construction.

### Biomedical Event Extraction

In the definition of ACE (Automatic Content Extraction) (Doddington et al., 2004), “event” is described as the occurrence of an action or the change of state. It includes an event trigger and multiple arguments with different roles. Event extraction is to obtain structured representations of events, so as to help answering the “5W1H” questions, including “who, when, where, what, why” and “how,” of real-world events from numerous sources of texts (Xiang and Wang, 2019). Biomedical event extraction is to extract events from biomedical texts and has become one of the most actively researched areas in biomedical text mining (Chung et al., 2020). Since the first biomedical natural language processing shared task (BioNLP-ST) challenge in 2009, the fine-grained biomedical event extraction has received extensive attentions from academia and industry.

Different from news (Abera, 2020), finance (Zheng et al., 2019) and other fields, biomedical event extraction has two main challenges. Firstly, biomedical texts have the higher complexity. They often contain many abbreviations and long sentences with complex structures. Secondly, high-quality annotated corpora are lacking. Most of the existing biomedical event corpora are small scale and have serious class imbalance problems. In recent years, a large number of studies have tried to solve these two problems. Various embedding technologies, such as dependency path embedding (Bjrne and Salakoski, 2018) and entity property embedding from external ontologies (Li et al., 2016), were developed to capture multi-aspect feature information for modeling complex biomedical texts. The hierarchical attention mechanism (Chen, 2019) was proposed to model the global document context for identifying biomedical event triggers from long biomedical sentences. Distance supervision (Araki and Mitamura, 2018), transfer learning (Chen, 2019), and other technologies have also been adopted to solve the problem of lack and imbalance of training samples.

In recent years, the knowledge base-oriented extraction task is becoming the research focus of biomedical event extraction. It evaluates the extraction systems or methods by measuring how much information content can be extracted from corpora (Deléger et al., 2016). Traditional pipeline models divided the event extraction task into multiple independent sub-tasks and resulted in various challenges, such as error propagation and lack of dependencies and interactions among subtasks. Therefore, the joint model becomes the inevitable choice of event extraction that oriented the knowledge base. Nguyen and Nguyen (2019) proposed a novel model to jointly perform predictions for entity mentions, event triggers, and arguments based on the shared hidden representations. Yu et al. (2019) developed an end-to-end model based on LSTM (Long Short-Term Memory) to optimize biomedical event extraction. Trieu et al. (2020) proposed an end-to-end neural nested event extraction model, named DeepEventMine, which can extract multiple overlapping directed acyclic graph structures from a raw sentence. Li et al. (2020) proposed a parallel multi-pooling convolutional neural network model to capture the compositional semantic features of sentences for improving biomedical event extraction on the MLEE (Multi-Level Event Extraction) dataset. Zhao et al. (2021) proposed a novel framework of reinforcement learning for the task of multiple biomedical event extraction, in which trigger identification and argument detection were treated as the main task and the subsidiary task.

Because of rich semantic information, the event is naturally more suitable to represent neuroimaging provenances, which characterize the research process of computational science, than isolated words. However, there are still two challenges in applying existing biomedical event extraction technologies, especially various joint models of biomedical event extraction, on research sharing-oriented neuroimaging provenance construction:

• Modeling the neuroimaging research process and result based on events. Existing biomedical event extraction tasks mainly focus on gene, protein, and disease-related events. As a new field of biomedical event extraction, neuroimaging event extraction lacks referential studies for task definition. Therefore, it is necessary to model the neuroimaging research process and results based on the perspective of events firstly. This is a challenging work because the definition of events needs to take into account both the demands of research sharing and the availability of knowledge in articles. The task type, content, and granularity of event extraction needs to be designed systematically by balancing various factors, including the task complexity, the capability of existing technologies, the importance and completeness of knowledge in research sharing, and the availability of knowledge in articles.

• Constructing the joint model of neuroimaging events in a few-shot learning scenario. Research sharing-oriented neuroimaging provenance construction is a typical task of knowledge base extraction and needs to adopt the joint model of event extraction. However, the complex network structure of joint model brings the higher requirements on the scale of training data. As a new field of event extraction, there are no existing annotated corpora for neuroimaging event extraction. Furthermore, neuroimaging event extraction in this study is involved with multi-domain entities for characterizing the research process of computational neuroscience. This also hinders the use of distance supervision (Araki and Mitamura, 2018) and transfer learning (Chen, 2019). Therefore, this is necessary to develop a new joint model of biomedical event extraction based on a small-scale “gold” corpus set annotated by domain experts.

## Contributions

The main contributions can be summarized as follows:

1. Firstly, this paper designs a provenance-guided approach for modeling the neuroimaging research process and result based on events. Considering the requirements of research sharing in open and FAIR neuroscience and availability of knowledge in neuroimaging articles, an improved brain informatics (BI) provenance model is defined based on NIDM. A mapping between the BI model and the definition of events is also proposed to obtain six categories of neuroimaging event-containing attributes for constructing research sharing-oriented neuroimaging provenances.

2. Secondly, this paper proposes a joint extraction model based on deep adversarial learning, called the adversarial training based neuroimaging event and attribute extraction model (AT-NeuroEAE), to extract the defined neuroimaging events. To the best of our knowledge, it is the first event extraction joint model containing attributes. Furthermore, the FreeAT (Free Adversarial Training)-based adversarial learning is introduced into the event joint extraction for improving the accuracy of event extraction in a few-shot learning scenario. This kind of optimization makes the proposed model more practical for computational neuroscience which lacks the “gold” labeled corpora.

3. Thirdly, a group of experiments were performed based on real data from the journal PLOS ONE. Experimental results show that, compared with existing event extraction models, the proposed model can more effectively and completely extract neuroimaging provenance information from neuroimaging articles based on a small-scale corpus set annotated by domain experts. This is a practical and effective approach for large-scale and low-cost neuroimaging provenance construction.

## Materials and Methods

### Overall Structure

**FIGURE 1:**
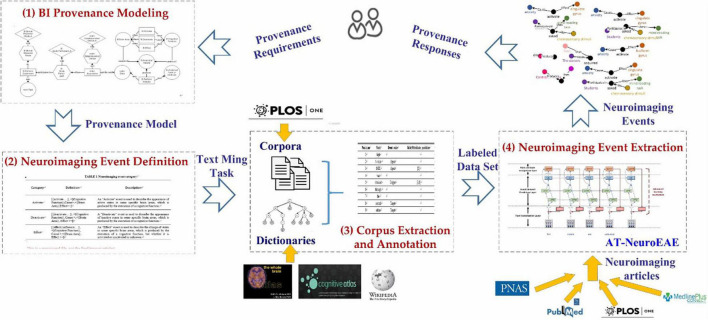
The whole process of provenance information extraction. (1) Brain informatics provenance modeling: construct an improved BI provenance model to capture the provenance requirements of research sharing in open and FAIR neuroscience. (2) Neuroimaging event definition: define a group of neuroimaging events to transform the BI provenance model into text mining tasks. (3) Corpus extraction and annotation: construct a group of labeled corpora for model training and test. (4) Neuroimaging event extraction: develop the NeuroEAE model to extract defined neuroimaging events for meeting the provenance requirements in open and FAIR neuroscience.

This paper proposes a joint extraction model of events with attributes, called AT-NeuroEAE, for research sharing-oriented neuroimaging provenance construction. By using NeuroEAE, provenance information can be automatically and quickly extracted from published neuroimaging articles, especially open-access scientific articles. As shown in Figure 1, the whole process includes four steps. Details will be described in the subsequent subsections.

### Brain Informatics Provenance Modeling

Computational neuroscience is a new application field of biomedical event extraction. In order to construct neuroimaging provenance by using biomedical event extraction, the first task needs to model the neuroimaging research process and result.

At present, NIDM is the most widely used neuroimaging provenance model and the representative work for open and FAIR Neuroscience (Abrams et al., 2021). However, as stated above, its element granularity is still incomplete for neuroimaging research sharing. It also contains a lot of model elements which aren’t available in articles. In our previous studies (Chen et al., 2012; Chen and Ning, 2013), a BI provenance model has been proposed to describe the origin and subsequent processing of various human brain data in systematic BI studies. This study reconstructs the BI provenance model by considering both the importance of knowledge in research sharing and the availability of knowledge in articles.

Firstly, the newest version of BI provenance model (Sheng et al., 2019) is updated based on the widely used NIDM. All entities, activities and agents are replaced by NIDM classes, such as “nidm:AcquisitionObject,” “nidm:Task,” “nidm:Acquisition,” and “nidm:DataAcquisitionDevice.” This update ensures that the new BI provenance model follows the FAIR facets I1: “(meta) data use a formal, accessible, shared, and broadly applicable language for knowledge representation” and I2: “(meta) data use vocabularies that follow FAIR principles.”

**FIGURE 2:**
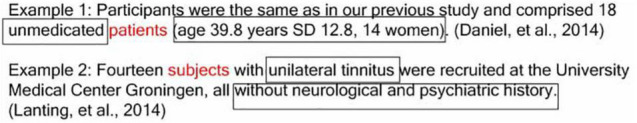
Diverse attributes of subjects in different articles. In example 1 (Daniel et al., 2014), the attributes of subjects include medication history, age, medical history, and gender. In example 2 (Lanting et al., 2014), the attributes of subjects include health condition and medical history.

Secondly, according to the FAIR facet F2, “data are described with rich metadata” and the deficiency of NIDM in experiment description; the abstract classes of experimental design in NIDM is extended as the activity “BI:PerformExperiment” and three related elements, including the entity “nidm:Task” and the entity “BI:StimuliResponseMode.” The entity “nidm:Task” and “nidm:StudyParticipant” are inherited from NIDM and used to indicate the task design type. The entity “BI:StimuliResponseMode” are used to describe the experiment in more detail. The former describes the sensory stimuli or response of experiment and the latter describes the participant of experiment. They are two important factors for cognitive experiments and need to be considered in multiple key steps of computational neuroscience, including experimental design, data analysis, result interpretation, etc. Neuroimaging articles also often introduce them in the “Experiment” section. Furthermore, both the stimuli mode and the subject are involved with multi-aspect attribute information, such as the perception channel, the stimuli task category, age, gender, and occupation, but it is not sure which and how much attribute information will be described in an article. As shown in Figure 2, different articles may describe subjects by using different attributes, including medication history, age, gender, medical history, and other complex characteristics. Therefore, this study adds the general attribute entity to represent all attributes of the entity. For example, the entity “BI:StudyParticipant_A” is added into the model to represent all possible attribute information of subjects. Similarly, according to the FAIR facet F2 “data are described with rich metadata” and the deficiency of NIDM in result description; this study adds three categories of activities “BI:Activate,” “BI:Deactivate,” “BI:Effect” and two related elements, including the entities “BI:BrainArea” and “BI:CognitiveFunction,” to describe the brain mechanism of cognitive states, which are core research findings in computational neuroscience.

Thirdly, considering both the importance and the availability of information, this study simplifies the description of NIDM about analytical tools or methods. NIDM contains many classes about parameters of analytical tools or methods. However, many parameters are generally not mentioned in the article. Therefore, this study uses an entity “BI:AnalyticalToolsorMethods,” which is connected to the activity “BI:PerformAnalysis,” to represent analytical tools or methods. Two entities “nidm:AcquisitionObject” and “BI:AnalyticalResults” are also connected to the activity “BI:PerformAnalysis” for representing the analytical process more clearly. Compared with the traditional class or concept tree, this kind of event-based description contains various roles within the event and can better describe the research process than the single subclass relation.

**FIGURE 3:**
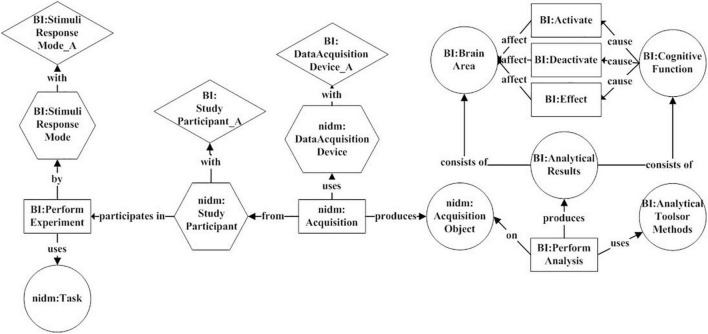
The BI provenance model. It uses six activities (rectangle), including “BI:PerformExperiment,” “nidm:Acquisition,” “BI:PerformAnalysis,” “BI:Activate,” “BI:Deactivate,” and “BI:Effect” to characterize experimental design, analytical process, and results. Six entities (circle), three agents (hexagon), and three attributes (diamond) are connected to these activities for describing various key factors in the research process.

Figure 3 gives the reconstructed BI provenance model. It characterizes experimental design, analytical process, and results by four categories of elements: entity, activity, agent, and attribute.

### Model-Guided Neuroimaging Event Definition

This study designs a model-guided approach to transform BI provenance model into text mining tasks. A group of neuroimaging events are defined by using the following mapping rules:

• Firstly, each activity and its directly connected elements (entities or agents) are divided into an independent activity unit.

• secondly, each activity unit is mapped to a meta event. The activity is mapped to the trigger words. The entities and agents are mapped to arguments. The relations between activity and elements are mapped to argument roles.

Based on the above rules, six categories of neuroimaging events can be obtained from the BI provenance model. Tables 1, 2 give the category definitions of events and corresponding arguments.

**TABLE 1:** Neuroimaging event category.

Category	Definition	Description
Activate	[{Activate… },<{cognitive function}, cause >,<{brain area}, affect > +]	An “Activate” event is used to describe the appearance of active states in some specific brain areas, which is produced by the execution of a cognitive function.
Deactivate	[{deactivate… }, < {Cognitive Function}, cause >,<{Brain Area}, affect > +]	A “Deactivate” event is used to describe the appearance of inactive states in some specific brain areas, which is produced by the execution of a cognitive function.
Effect	[{effect | influence… }, < {Cognitive Function}, cause >, < {Brain Area}, affect > +]	An “Effect” event is used to describe the change of states in some specific brain areas, which is produced by the execution of a cognitive function, but whether it is activated or inactivated is unknown.
Perform experiment	[{perform | complete… }, < {Study Participant (Study Participant_A)*}, participates in > *, < {Task}, uses > +, < {Stimuli Response Mode (Stimuli Response Mode_A)*}*, by >]	A “Perform Experiment” event is used to describe a neuroimaging research action by a group of study participants, i.e., subjects do one or several experimental tasks by some kind of stimuli response mode. Study participants often have some attributes, such as age, gender, and medical history. The stimuli response mode is also involved with some features, such as the perception channel, the stimuli task category.
Acquisition	[{assess | acquire | obtain…}, < {Acquisition Object}, produces > *, < {Data Acquisition Device (Data Acquisition Device_A)*}, uses > +, < {Study Participant (Study Participant_A)*}, from > *]	The “Acquisition” event is used to describe a research action that a data acquisition device with relevant parameters produces physiological and psychological data from study participants.
Perform analysis	[{perform | complete… }, < {Analytical Results}, produces > +, < {Acquisition Object}, on > *, < {Analytical Tools or Methods}, uses > +]	The “Perform Analysis” event is used to describe a research action that the analytical tool or method is used on physiological and psychological data to produce a group of analytical results, i.e., brain responses, such as Default Mode Network (dmn).

“*” indicates that the element may occur zero or more times and “+” indicates that the element may occur one or more times.

**TABLE 2:** Argument categories.

Argument category	Description
Cognitive function (COG)	The cognitive function is an ability of the human brain to process information and used to denote the brain function implied by brain responses in computational neuroscience research.
Brain area (BRI)	The brain area is an anatomical region of the cerebral cortex and used to mark the occurrence location of brain response in computational neuroscience research.
Data acquisition device (ACQ)	The data acquisition device is a kind of brain testing equipment used in computational neuroscience research.
Stimuli response mode (SEN)	The stimuli response mode is used to denote the sensory channel of stimulus presentation in computational neuroscience research.
Study participant (STP)	The study participant is a participant in computational neuroscience research and recorded for behavioral or brain physiological data.
Task (TSK)	The experimental task is a task (e.g., questions, games, etc.) which is performed by the study participant in computational neuroscience research.
Acquisition object (AOB)	The acquisition object is a kind of physiological and psychological data which are collected by the data acquisition device in computational neuroscience research.
Analytical tools or methods (TOL)	The analytical tool or methods is a mining algorithm or tool which is used to analyze experimental data in computational neuroscience research.
Analytical results (RLT)	The analytical results are a series of brain responses which are mined from experimental data in computational neuroscience research.

In this study, the definition of events is represented as trigger + parameter structure (Sun et al., 2017). For example, an “Acquisition” event can be represented as follows:

Event Acquisition

= [trigger, < argument1(attribute)#, role1 > #, < argument2(attribute)#, role2 > #…]

= [{assess | acquire | obtain…}, < {Acquisition Object}, produces > *, < {Data Acquisition Device (Data Acquisition Device_A)*}, uses > +, < {Study Participant (Study Participant _A)*}*, from > ]

**FIGURE 4:**
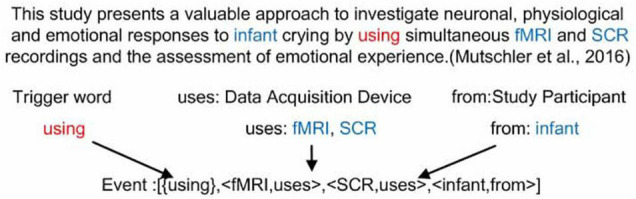
An example of an “Acquisition” event from the article (Mutschler et al., 2016). This event consists of a trigger word “using,” two “Data Acquisition Device” category of arguments “fMRI” and “SCR,” and one “Study Participant” category of argument “infant.”

The above expression shows that an “Acquisition” event consists of a trigger word and three categories of arguments. “*” indicates that the element may occur zero or more times and “+” indicates that the element may occur one or more times. The trigger word may be one of “assess,” “acquire,” “obtain,” etc. Argument 1 belongs to the “Acquisition Object” category and is used to describe the results produced by the “Acquisition” event. Argument 2 belongs to the “Data Acquisition Device” category and is used to describe the device used in the “Acquisition” event. Argument 3 belongs to the “Study Participant” category and is used to describe the object of the “Acquisition” event. Both Argument 2 and Argument 3 have zero or several attributes. Figure 4 gives an example of an “Acquisition” event in a neuroimaging article.

### Corpus Extraction and Annotation

Corpora were crawled from the journal PLOS ONE. We searched the articles based on the keywords “fMRI” or “functional magnetic resonance imaging” or “functional MRI” in their abstracts, and set the published time from July 18, 2014, to July 18, 2019. A total of 677 articles were obtained. Based on these articles, event mentions were extracted by using rule matching. The process can be described as follows.

The first step is dictionary construction. Seven categories of argument dictionaries, including “Brain Area,” “Cognitive Function,” “Study Participant,” “Stimuli Response Mode,” “Task,” “Analytical Tools or Method,” and “Analytical Results” came from our previous study (Sheng et al., 2020). We collected candidate trigger words and arguments based on the co-occurrence with these seven categories of arguments, and then manually filtered them to construct the trigger word dictionaries and other categories of argument dictionaries. Similarly, three categories of argument attribute dictionaries were constructed based on the co-occurrence and manual filtering. Table 3 gives the number of terms in each dictionary.

**TABLE 3:** The number of terms in dictionaries.

Element type	Element	Term number	Element	Term number
Trigger word	Acquisition	65	Effect	145
	Perform experiment	62	Deactivate	20
	Perform analysis	227	Activate	12
Argument	BRI	576	SEN	52
	COG	812	STP	97
	AOB	48	TOL	171
	ACQ	57	TSK	814
	RLT	28		
Argument	Data acquisition device_A	33	Study participant_A	7
	Stimuli response mode_A	24		

The second step is mention extraction. Based on the dictionaries and event definitions, event mentions were extracted by using the following rule:

*Rule1: If an event trigger word and two arguments appear in the same sentence, this sentence can be marked as a complete event.*

However, Rule 1 cannot extract enough the “Deactivate” event. We defined another more relaxed rule:

*Rule2: If a “Deactivate” event trigger word and a “BRI” argument appear in the same sentence, this sentence can be marked as a “Deactivate” event.*

**FIGURE 5:**
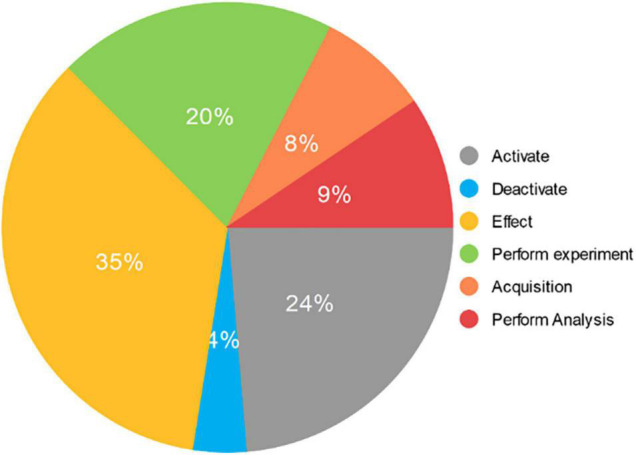
The distribution of event mentions in the experimental data set. It consists of 3331 event mentions extracted from 677 neuroimaging articles. The “Activate” category includes 788 mentions and accounts for 24% of the total. The “Deactivate” category includes 128 mentions and accounts for 4% of the total. The “Effect” category includes 1169 mentions and accounts for 35% of the total. The “Perform experiment” category includes 665 mentions and accounts for 20% of the total. The “Acquisition” category includes 266 mentions and accounts for 8% of the total. The “Perform Analysis” category includes 315 mentions and accounts for 9% of the total.

Based on Rules 1 and 2, a neuroimaging event mention set can be obtained as the experimental data set. It includes 788 “Activate” event mentions, 128 “Deactivate” event mentions, 1169 “Effect” event mentions, 665 “Perform Experiment” event mentions, 266 “Acquisition” event mentions, and 315 “Perform Analysis” event mentions. The distribution of event mentions in the experimental data set is shown in Figure 5.

In the training dataset, the annotation of triggers and arguments adopts the “B/I/O-category abbreviation” mode. The “B/I/O” is the BIO annotation mode (Soomro et al., 2017). “B” indicates the beginning of entity. “I” stands for the middle or end, and “O” stands for other, which is used to mark irrelevant characters. Category abbreviations are involved with argument categories and trigger categories. The entity annotation of attributes adopts the “B/I/O-category abbreviation_A” model. Table 4 gives an annotation example. The labels “BRI” and “AOB” indicate the argument categories “Brain Area” and “Acquisition Object,” respectively. The label “Acq” indicates the trigger category “Acquisition.” The label “O” on the word “high” indicates “high” does not belong to any entity. The label “B-AOB” on the word “fMRI” indicates “fMRI” is the beginning of the “Acquisition Object” argument.

**TABLE 4:** Event element annotation.

Annotation example								
High	*b*-value	fMRI	Was	Obtained	Through	The	Central	Sulcus
O	O	B-AOB	O	B-Acq	O	O	B-BRI	I-BRI

The annotation of event roles and attributes is based on trigger words. As shown in Table 5, the first column is the position and the second column is the corresponding word. If the word is a trigger word, its third column is marked “Trigger” and its fourth column is marked with the form of “[ArgPos_1_, ArgPos_2_,…, ArgPos_*n*_,…]” in which “ArgPos_*n*_” is the starting position of the corresponding argument in role_*n*_. If the word is an argument, its third column is marked by the corresponding role category label. And if this argument has attributes, its fourth column is marked with the form of “[AttPos_1_, AttPos_2_,…, AttPos_*n*_,…]” in which “AttPos_*n*_” is the starting position of its attribute_*n*_.

**TABLE 5:** Event role and attribute annotation.

Position	Word	Event role	Role/attribute position
1	High		
2	*b*-value	Produces	
3	fMRI	Produces	[2]
4	Was		
5	Obtained	Trigger	[3,8]
6	Through		
7	The		
8	Central	From	
9	Sulcus	From	

### Neuroimaging Event Extraction Based on AT-NeuroEAE

**FIGURE 6:**
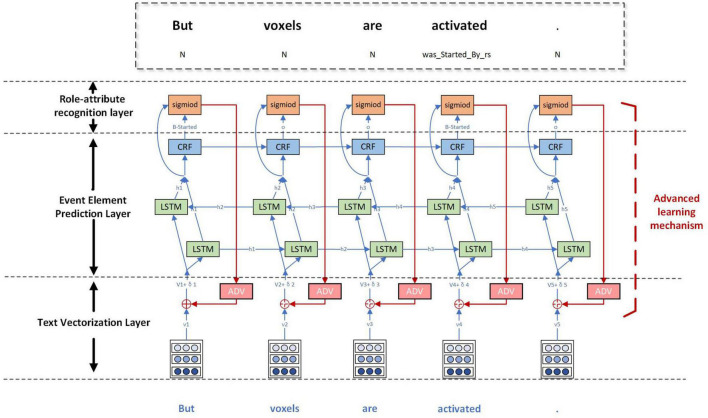
The AT-NeuroEAE model. The text vectorization layer encodes sentences as textual vectors based on lexical units, case features, and domain terminology dictionaries. The event element prediction layer predicts the potential event elements by using the BiLSTM-CRF model. The role-attribute recognition layer identifies the role and attribute of argument by using the sigmoid function. The adversarial learning mechanism adds small and persistent disturbances to the input of joint model for improving the robustness and generalization of the model. BiLSTM: bi-directional long short-term memory; CRF: conditional random fields; adv: adversarial learning.

In order to realize the event extraction containing attributes, this study proposes a joint event extraction model based on deep adversarial learning, called AT-NeuroEAE. The multi-layer joint extraction model of neuroimaging events with attributes is constructed to synchronously realize the prediction of event elements, including trigger, arguments, and attributes and the extraction of element relations, including argument roles and argument attributes, by the end-to-end mode. The adversarial learning based on FreeAT is combined with this joint model to realize the event extraction in a few-shot learning scenario. Figure 6 gives the whole structure of the model, which consists of the text vectorization layer, the event element prediction layer, the role-attribute recognition layer, and the adversarial learning mechanism.

In this experiment, we set the dimension of the word vector to 25, the number of iterations to 200, the dimension of the LSTM hidden layer to 64, the number of LSTM layers to 3, the dropout to 0.9, the learning rate to 1e-3, the activation function to tanh, and the adversarial disturbance parameter alpha to 0.01. Parameter optimization used the Adam algorithms.

#### Text Vectorization Layer

The text vectorization layer encodes sentences as textual vectors for the input of model. Suppose that there is a sentence *s* = [w_1_, w_2_, … w_*n*_]. For each word w_*i*_, three categories of feature vectors are constructed based on lexical units, case features, and domain terminology dictionaries, respectively.

• Word vector. The word vector is learned to contain as much semantic and grammatical information as possible (Pennington et al., 2014). This study adopts the Glove word vector model which was trained on 6 billion words of Wikipedia and web texts (Pennington et al., 2014).

• Case feature vector. Neuroimaging event mentions are involved with a large number of domain terms with capitalized abbreviated names, such as fMRI. In order to capture this kind of corpus features, this study constructs a one-hot case feature vector including six dimensions “numeric,” “allLower,” “allUpper,” “initialUpper,” “mainly_numeric,” “contains_digit,” and “other.”

• Terminology dictionary vector. Neuroimaging event elements involve nine types of important domain entities shown in Table 2. This study constructs the one-dimensional terminology dictionary vector (Xu et al., 2019) to capture this kind of domain corpus features. Nine term dictionaries are collected for these nine categories of domain entities firstly, and then a label list is constructed base on the “B/I/O-entity category abbreviation.” When w_*i*_ matches any term in the dictionaries, a label index is set as the dimensional value of terminology dictionary vector of w_*i*_.

Concatenating these three categories of feature vectors (Zhang et al., 2019), the combined word vector of w_*i*_ can be obtained as follows:


(1) $${\text{v}}_{i}=\text{[}v_{w},v_{c},v_{t}]$$

where *v_w_*, *v*_*c*_, and *v_t_* are the corresponding word vector, case feature vector, and terminology dictionary vector of w_*i*_.

#### Event Element Prediction Layer

The event element prediction layer predicts the potential event elements (i.e., argument, attribute, or trigger word) by using the BiLSTM-CRF model. BiLSTM is used to model context information of sentences based on input text vectors. The process can be defined as follows:


(2) $$h_{i}=\left[\vec{LSTM}\left(v_{i}\right),\overset{\leftarrow }{LSTM}\left(v_{i}\right)\right],i\in \left[1,n\right]$$

where, *v_i_* is the combination vector of *w_i_*, $\vec{LSTM}$ is the forward hidden layer output of LSTM, $\overset{\leftarrow }{LSTM}$ is the backward hidden layer output of LSTM, and *h_i_* is a word representation of each word token.

CRF is used to predict the element category and boundary. Event element prediction is a named entity recognition task which can be formulated as a sequence labeling problem. Based on the BIO annotation mode, each event element often consists of multiple sequential tokens. We calculate the score of each token *w_i_* for each event element tag:


(3) $$S^{\left(e\right)}\left(h_{i}\right)=V^{\left(e\right)}f\left(U^{\left(e\right)}h_{i}\right),i\in \left[1,n\right]$$

where the superscript (e) means event element prediction; *f*(⋅) is the activation function; *V*^(e)^ ∈ ℝ^p = *l*^, *U*^(e)^ ∈ ℝ^2*d* = *l*^, and *l* are the width of LSTM layer; and *d* is the hidden size of LSTM layer. The linear-chain CRF score of the *s* is defined as:


(4) S(y1(e),y2(e),…,yn(e))=∑i=0nsi,yi(e)(e)+∑i=1n-1Tyi(e),yi+1(e),i∈[1,n]

where *y*_*i*_ ∈ Y is the tag of *w_i_*, *s*_*i,y_i_*_ is the predicted score of *w_i_* given a tag *y_i_*, and T is the transition matrix.

Then the probability of a given tag sequence on all possible tag sequences of the input sentence *sen* is defined as:


(5) Pr=(y1(e),y2(e),…,yn(e)|sen)eS(y1(e),y2(e),…,yn(e))∑y1~(e),…,yn~(e)es(y1~(e),…,yn~(e))

In the process of training, this study minimizes the cross-entry loss ℒ_*NER*_:


(6) $${ℒ}_{NER}=\sum_{i=0}^{n}-logPr\left(y_{i}|w_{i};\theta \right)$$

#### Role-Attribute Recognition

The role-attribute recognition layer identifies the role and attribute of argument by using the sigmoid function. Recognizing the attribute of argument is the one-to-one relation extraction between the argument and its attribute, but recognizing the role of argument is the one-to-n relations because the trigger word is usually corresponding to multiple arguments in an event. Hence, this paper models the role recognition task as a multi-label head selection problem (Zheng et al., 2017), i.e., the relation extraction between one trigger word and two or more arguments.

As shown in Figure 4, the role-attribute recognition layer receives the hidden state of LSTM from the event element prediction layer. The predication score between tokens *w_i_* and *w_j_* given a role-attribute tag *r_k_* can be calculated as follows:


(7) $$s^{\left(r\right)}\left(h_{i},h_{j},r_{k}\right)=V^{\left(r\right)}f\left(U^{\left(r\right)}h_{i}+W^{\left(r\right)}h_{j}\right)$$

where the superscript (*r*) means role-attribute recognition, *f*(⋅) is the activation function, *h*_*i*_ is the output of the hidden state of LSTM corresponding to *w_i_*, *V*^(*r*)^ ∈ ℝ^*l*^ *l* is the width of the LSTM layer, *U*^(*r*)^ ∈ ℝ^l*2*d*^, *W*^(*r*)^ ∈ ℝ^l*2*d*^, and *d* is the hidden size of LSTM layer. The probability of token *w_j_* to be selected as the head of token *w_i_* with the role-attribute tag *r_k_* between them can be calculated as follows:


(8) $$Pr\left(head=w_{j},tag=r_{k}|w_{i},\theta \right)=\sigma \left(s\left(m_{j},m_{i},r_{k}\right)\right)$$

where θ is a set of parameters and σ(⋅) is the sigmoid function.

In the process of training, this paper minimizes the cross-entry loss ℒ_*RAR*_:


(9) $${ℒ}_{RAR}=\sum_{i=0}^{n}\sum_{j=0}^{m}-Pr\left(head=y_{i,j},relation=r_{k}|w_{i}\right)$$

where *y_i_* is the ground truth vector and *r_k_* is the role-attribute tag, and *y*_*i*_⊆ *sen* and *r_k_* ⊆ R and *m* (*m* < *n*) are the number of associated heads (i.e., roles or attributes) of *w_i_*. During decoding, the most probable heads and roles/attributes are selected using threshold-based prediction.

Finally, for the joint event extraction task, the final objective is calculated as ℒ_*NER*_ + ℒ_*RAR*_. This study minimizes this objective function in the process of training of joint model.

#### Adversarial Learning Mechanism

The adversarial learning mechanism adds small and persistent disturbances to the input of the joint model. After the adversarial training with disturbances, the output distribution is consistent with the original distribution, but the robustness and generalization of the model can be improved (Wang et al., 2018).

This study adopts the perturbation strategy of FreeAT proposed by Shafahi et al. (2019). As shown in Figure 5, repeat K times for each sample continuously to find the optimal perturbation. The gradient of the previous step is multiplexed when calculating the perturbation. Finally, the overall iteration is divided by K to greatly reduce the speed problem caused by the inner iteration. The approximation of the perturbation is defined as:


(10) $$r_{advt+1}=r_{advt}+\in \frac{\nabla_{w}ℒ\left(w;\hat{\theta }\right)}{||\nabla_{w}ℒ\left(w;\hat{\theta }\right)||}$$

where, *r*_adν_ is the perturbation, $\hat{\theta }$ is a copy of the current model parameter, *t* ⊆ K, ∈$=\alpha \sqrt{D}$ is a hyperparameter, α is the factor and *D* is the dimension of the embeddings *v*_*i*_, and *t* is the number of inner iterations.

Finally combining the original examples and the confrontation examples for model adversarial training, the final loss of joint model is as follows:


(11) $$ℒ\left(w,\hat{\theta }\right)={ℒ}_{joint}\left(w;\hat{\theta }\right)+{ℒ}_{joint}\left(w+r_{adv};\hat{\theta }\right)$$

where $\hat{\theta }$ is the current value of the model parameter.

### Evaluation

The precision rate *P*, the recall rate *R*, and the *F*_*1*_ value (Curiskis et al., 2019) are adopted to evaluate experimental results. They can be calculated as follows:


(12) $$P=\frac{\left|TP\right|}{\left|TP\right|+\left|FP\right|}$$

(13) $$R=\frac{\left|TP\right|}{\left|TP\right|+\left|FN\right|}$$

(14) $$F_{1}=\frac{2\times Precision\times Recall}{Precision+Recall}$$

In this study, *P*, *R*, and *F*_*1*_ values are used to evaluate the results of subtasks, but only *F*_*1*_ values are used to evaluate the overall results of event extraction.

## Results

### Baseline Methods

The CNN-BiLSTM-PCNN (Convolutional Neural Network-Bi-directional Long Short-Term Memory-Pulse Coupled Neural Network) pipeline model (Zheng et al., 2017) and the GCN (Graph convolution Network) joint model^9^ are used in the two control experiments. The CNN-BiLSTM-CNN pipeline model uses the CNN-BiLSTM model for the subtask of event element prediction, and the PCNN model for the subtask of role attribute recognition.

In many competitions, these models have been proved to be stable and relatively optimal for various biomedical named entity recognition tasks and relation extraction tasks. The GCN joint model was used on multiple shared tasks of biomedical event extraction, including BB3 (Bacteria Biotope) event data set (Lever and Jones, 2016), and SeeDev (Plant Seed Development) event data set (Agirre et al., 2019), and has obtained good experimental results.

In this study, these two models used their original parameters firstly, and then we adjusted parameters referring to some classic articles (Kip and Welling, 2016; Ma and Hovy, 2016; Zhang et al., 2019). Based on the results, a group of optimal parameters were chosen as follows:

• The CNN-BILSTM-PCNN pipeline model: For the event element prediction subtask, the dimension size of the word vector was set at 100. The epoch number was set at 50, the convolution width at 3, the CNN output size at 30, the dimensional number of LSTM hidden layer at 200, the mini-batch size at 9, and the dropout at 0.5. The Adam algorithm was used to optimize parameters. For the role-attribute recognition subtask, the dimension size of the word vector was set at 50, the convolution width at 3, the dimensions of the two hidden layers at 200 and 100, respectively, and the learning rate at 0.01.

• The GCN joint model: The dimension size of the word vector was set at 300, the dimension size of the Hidden layer at 200, the epoch number at 50, dropout of GCN at 0.5, dropout of word at 0.5, and the batch size at 16.

### Results Analysis

This study adapted fivefold cross validation to improve the objectivity of results. Each category of event mentions was randomly divided into five equal parts. In order to simulate the few-shot learning scenario, each experiment used one part as the training data set and the remaining four parts as the test data set. After five experiments, the average results were taken as the final results.

Table 6 gives F_1_ values of all models. It can be seen that the CNN-BiLSTM-PCNN pipeline event model lags behind the other three models in five categories of events except “Acquisition.” This shows the necessity of joint model in the complex text mining task, such as event extraction. The proposed NeuroEAE model is superior to the GCN joint model in all event categories except the “Analytical results” event. This proves the validity of proposed model structure for extracting neuroimaging event containing attributes.

**TABLE 6:** F_1_ values of event extraction.

Event category	CNN-BiLSTM-PCNN	GCN	NeuroEAE	AT-NeuroEAE
Acquisition	0.588	0.542	0.689	0.734
Perform analysis	0.448	0.596	0.547	0.671
Perform experiment	0.368	0.429	0.612	0.853
Effect	0.569	0.576	0.690	0.692
Activate	0.614	0.682	0.809	0.756
Deactivate	0.922	0.952	0.974	0.608

The proposed AT-NeuroEAE model introduces adversarial learning into NeuroEAE. It achieved the better results on most of event categories than other models. This shows the validity of adversarial learning in the few-shot learning scenario. However, AT-NeuroEAE loses to NeuroEAE on the “Activate” and “Deactivate” events. This may be due to the fact that its structure and elements are relatively single compared with other event categories. As the conclusion of computational neuroscience research, the “Activate” and “Deactivate” events are often described by using the simple and clear languages in articles. For example, “We previously showed that a common network of brain regions was activated for past and future thinking in healthy older adults” (Rodolphe et al., 2014) and “This cortical area was found to be activated in studies where participants had to perform a task of face vs. non-face recognition” (Prehn-Kristensen et al., 2009). Under the same degree of adversarial disturbance of the model, such a simple event structure is easy to produce the collapse problem, which makes the model unable to further learn. This shows that the increasing disturbance can lead to the reduction of model accuracy under the simple event extraction. For other event categories, whether it is the “Analytical Results” event with the complex structure or the “Effect” event with more trigger word instances, the model has advantages by using adversarial learning. Hence, the proposed AT-NeuroEAE is an effective model to extract neuroimaging events from complex event mentions, which are common in neuroimaging articles. If the event is simpler, the NeuroEAE model is the better choice. Extracting neuroimaging event-containing attributes can be realized by flexibly using these two kinds of models.

Table 7 gives the F_1_ values of each experiment in fivefold cross validation. As shown in this table, the results are stable in each experiment. Our experiments were performed on the personal computer with CPU Intel Core 9th Generation i7 and Graphics Card NVIDIA GeForce GTX 1650. The epoch number was set at 100. Table 8 gives the running time of each experiment. The average running time of model training is almost 4.8 h and the average running time of model test is almost 6.7 s. This shows that, although the model training takes a long time, the speed of event extraction is still relatively fast and can meet the requirements of rapid extraction of provenances.

**TABLE 7:** F_1_ values in fivefold cross validation.

Event category	*F*_1_-values					
	1	2	3	4	5	Average
Acquisition	0.700	0.720	0.768	0.763	0.722	0.734
Perform analysis	0.676	0.674	0.699	0.610	0.696	0.671
Perform experiment	0.889	0.837	0.863	0.871	0.808	0.853
Effect	0.724	0.686	0.696	0.734	0.618	0.692
Activate	0.772	0.756	0.740	0.781	0.728	0.756
Deactivate	0.639	0.613	0.608	0.600	0.583	0.608

**TABLE 8:** Running times in fivefold cross validation.

Experiment	1	2	3	4	5	Average
Model training (s)	17194.8	17601.7	17331.0	17425.7	17191.0	17348.8
Model test (s)	7.46	5.95	6.79	6.98	6.18	6.7

## Discussion

### Results Analysis on Subtasks

Neuroimaging event extraction consists of two subtasks: event element prediction and role-attribute recognition. This section will analyze results of each subtask separately to understand and evaluate the proposed AT-NeuroEAE model in depth.

Table 9 gives the results of event element prediction, which are involved with three kinds of elements, i.e., trigger words, arguments, and attributes. The full names of arguments are mentioned in Table 2. The “abbreviation_A,” such as DAT_A, denotes the attribute of the corresponding argument. In this study, the prediction task of attribute is most ambiguous because the general attribute indicates all of possible actual attributes, as stated above. The prediction task of arguments is clearly defined but each argument category is often involved with a large number of entity instances. The prediction task of trigger words is clearest and simplest. Each category of trigger words only involved limited words. Comparing prediction results on these three kinds of elements, we can see that the clearer and simpler the task, the better the result. Generally, all elements are recognized well. F_1_ values of the AT-NeuroEAE model are almost above 0.9. Even the worst model CNN-BiLSTM-PCNN, its F_1_ values on all categories of elements are more than 0.7. This shows that the existing deep learning models have been able to effectively solve the task of named entity recognition, as stated in Armelle et al. (2014).

**TABLE 9:** Event element prediction.

Element		CNN-BiLSTM-PCNN			NeuroEAE			AT- NeuroEAE		
		*P*	*R*	*F*_1_	*P*	*R*	*F*_1_	*P*	*R*	*F*_1_
Trigger word	Acquisition	0.864	0.888	0.876	0.951	0.925	0.938	0.972	0.972	0.972
	Perform experiment	0.841	0.803	0.821	0.851	0.802	0.826	0.915	0.915	0.915
	Analytical results	0.879	0.79	0.832	0.981	0.879	0.927	0.843	0.931	0.885
	Effect	0.958	0.846	0.898	0.956	0.945	0.951	0.951	0.951	0.951
	Deactivate	0.821	1	0.902	1	0.958	0.978	1	1	1
	Activate	1	0.985	0.993	0.889	1	0.941	0.971	0.985	0.978
Argument	BRI	0.863	0.871	0.867	0.929	0.908	0.919	0.921	0.908	0.915
	COG	0.744	0.703	0.723	0.769	0.704	0.735	0.861	0.789	0.824
	AOB	0.941	0.899	0.92	0.975	0.91	0.941	0.988	0.943	0.965
	ACQ	0.962	0.962	0.962	0.897	1	0.897	1	1	1
	RLT	0.963	0.897	0.929	1	0.896	0.945	0.964	0.931	0.947
	SEN	0.972	0.648	0.778	0.895	0.781	0.834	0.959	0.854	0.903
	STP	0.744	0.727	0.736	0.851	0.909	0.879	0.811	0.977	0.886
	TOL	0.776	0.844	0.809	0.864	0.711	0.78	0.907	0.867	0.886
	TSK	0.982	0.873	0.924	0.936	0.936	0.937	0.953	0.968	0.961
Argument attribute	ACQ_A	0.667	0.905	0.776	0.944	0.809	0.871	0.9	0.857	0.878
	SEN_A	0.744	0.806	0.773	0.794	0.75	0.771	0.965	0.778	0.862
	STP_A	0.962	0.833	0.893	0.964	0.9	0.931	1	0.966	0.983

Comparing AT-NeuroEAE and NeuroEAE, most F_1_ values are improved and proved the effectiveness of adversarial learning. The negative effects of adversarial learning mainly appear in attribute prediction. This shows that the ambiguous task definition may not be suitable for adversarial learning.

Table 10 gives the results of role-attribute recognition, which are involved with two aspects of relations, i.e., element roles and element-attribute relations. Obviously, adversarial learning improves the F_1_ values of role-attribute recognition for the majority of roles and attributes. The F_1_ value of “Analysis-Agent” role is significantly improved after adding adversarial learning. The main reason is that the corresponding “Perform Analysis” event has the complex structure. This confirms the above judgment that the adversarial learning is more suitable for complex tasks.

**TABLE 10:** Role-attribute recognition.

Event role	CNN-BiLSTM-PCNN			NeuroEAE			AT- NeuroEAE		
	*P*	*R*	*F*_1_	*P*	*R*	*F*_1_	*P*	*R*	*F*_1_
Acquisition-uses	0.597	0.74	0.661	0.708	0.667	0.687	0.766	0.706	0.735
Acquisition-produces	0.558	0.898	0.688	0.772	0.809	0.791	0.8	0.762	0.781
Perform analysis-on	0.571	0.667	0.615	0.681	0.687	0.684	0.729	0.794	0.761
Perform analysis-produces	0.5	0.692	0.581	0.686	0.528	0.597	0.687	0.632	0.658
Perform analysis-uses	0.7	0.333	0.451	0.667	0.5	0.571	0.642	0.843	0.729
Perform experiment-by	0.471	0.696	0.562	0.56	0.583	0.571	0.619	0.541	0.578
Perform experiment-participates in	0.3	0.462	0.364	0.416	0.357	0.384	0.462	0.428	0.444
Perform experiment-uses	0.511	0.649	0.571	0.56	0.736	0.636	0.591	0.684	0.634
Effect-affect	0.75	0.788	0.768	0.711	0.821	0.762	0.832	0.806	0.818
Effect-cause	0.649	0.649	0.649	0.677	0.677	0.677	0.731	0.686	0.708
Deactivate-affect	0.875	0.966	0.918	1	1	1	1	1	1
Deactivate-cause	0.905	0.95	0.918	0.952	0.952	0.952	1	1	1
Activate-affect	0.862	0.812	0.836	0.833	0.864	0.848	0.861	0.765	0.81
Activate-cause	0.566	0.625	0.594	0.676	0.588	0.625	0.667	0.549	0.602
ACQ-attribute	0.594	0.92	0.867	0.956	0.709	0.814	0.806	0.806	0.806
SEN-attribute	0.385	0.385	0.385	1	0.526	0.689	0.9	0.346	0.5
STP-attribute	1	0.815	0.898	0.692	0.62	0.654	0.772	0.586	0.667

In addition, we can see that the F_1_ values of the “Activate-affect” role and “Activate-cause” role in the “Activate” event decrease after adding adversarial learning. As stated above, the “Activate” event often has the simple and clear language structure because it is the research conclusion. Table 9 shows that the F_1_ values of the element “Brain Area” (BRI) in this event also decrease after adding adversarial learning. This confirms the above judgment again that the adversarial learning is not suitable for simple tasks. The F_1_ values of another event element “Cognitive Function” (COG) don’t decrease mainly because “Cognitive Function” is most complex element category and has thousands of entity instances (Poldrack et al., 2011) in different forms. The F_1_ values of two categories of attribute relations “ACQ-Attribute” and “SEN-Attribute” also decrease after adding adversarial learning. This confirms the above judgment again that adversarial learning is not suitable for ambiguous tasks.

Comparing Tables 9, 10, we can see that in the first subtask, the results of CNN-BiLSTM-PCNN pipeline model are similar to our two joint models, but there is a big gap in the second subtask. The reason is the existence of cascading errors. As shown in Table 11, when cascading errors are removed manually, CNN-BiLSTM-PCNN can also achieve good results. This proves the validity of joint models.

**TABLE 11:** Comparison of pipeline models with and without cascading errors.

Event role	CNN-BiLSTM-PCNN (no cascading errors)			CNN-BiLSTM-PCNN		
	*P*	*R*	*F*_1_	*P*	*R*	*F*_1_
Acquisition-uses	0.657	0.98	0.951	0.597	0.74	0.661
Acquisition-produces	0.612	1	0.759	0.558	0.898	0.688
Perform analysis-on	0.695	0.842	0.761	0.571	0.667	0.615
Perform analysis-produces	0.692	0.857	0.765	0.5	0.692	0.581
Perform analysis-uses	1	0.478	0.647	0.7	0.333	0.451
Perform experiment-by	0.469	0.958	0.63	0.471	0.696	0.562
Perform experiment-participates in	0.423	0.785	0.55	0.3	0.462	0.364
Perform experiment-uses	0.692	0.947	0.799	0.511	0.649	0.571
Effect-affect	0.886	0.921	0.903	0.75	0.788	0.768
Effect-cause	0.783	0.775	0.779	0.649	0.649	0.649
Deactivate-affect	0.906	1	0.951	0.875	0.966	0.918
Deactivate-cause	0.869	1	0.93	0.905	0.95	0.918
Activate-affect	0.975	0.962	0.968	0.862	0.812	0.836
Activate-cause	0.833	0.882	0.857	0.566	0.625	0.594
ACQ-attribute	0.878	0.935	0.906	0.594	0.92	0.867
SEN-attribute	0.96	0.923	0.941	0.385	0.385	0.385
STP-attribute	0.96	0.827	0.889	1	0.815	0.898

### Result Comparison With Existing Studies on Neuroimaging Text Mining

As stated in section “Neuroimaging Text Mining,” existing studies on neuroimaging text mining can be divided into neuroimaging topic modeling and neuroimaging named entity recognition. This section will compare the proposed AT-NeuroEAE with these two kinds of neuroimaging text mining, respectively.

**FIGURE 7:**
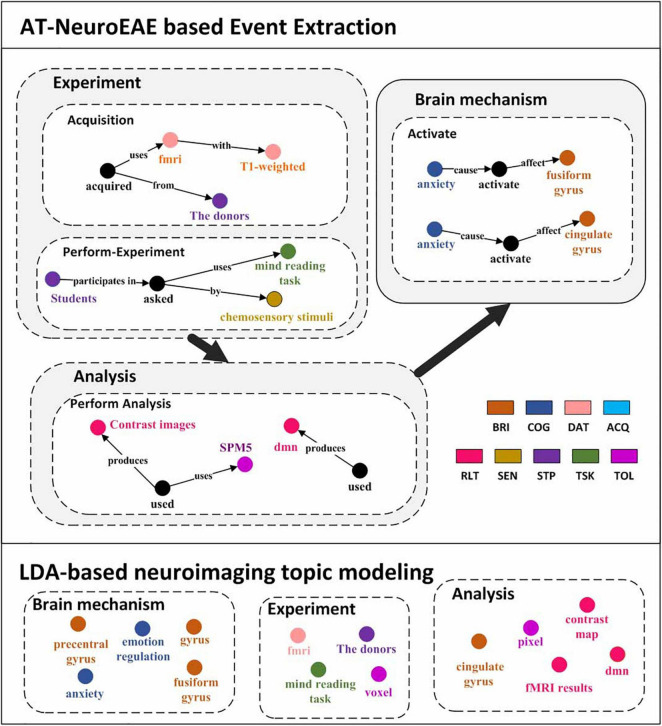
Visualization results of event extraction. The top is five events extracted from the article (Prehn-Kristensen et al., 2009). The bottom is LDA topics extracted from the same article. In order to compare with the results of AT-NeuroEAE, LDA topics are manually divided into three classes, brain mechanism, experiment, and analysis.

LDA is the most commonly used method for neuroimaging topic modeling and has been applied to many previous studies (French et al., 2012; Alhazmi et al., 2018). Different from quantitative analysis based on P, R, and F_1_, this section compares extracted contents between the proposed AT-NeuroEAE and LDA-based neuroimaging topic modeling by using a specific article. Figure 7 shows the results of AT-NeuroEAE and LDA for the article titled “Induction of Empathy by the Smell of Anxiety” (Prehn-Kristensen et al., 2009). Different colors represent different categories of entities or topics. The random state was set to 1, the number of topics to 15, and alpha to 0.5.

As shown in Figure 7, LDA can obtain multi-aspect topics, which are involved with brain mechanism (e.g., anxiety, frontal gyrus), the experiment (e.g., mind reading task), and analysis (e.g., voxel). However, these topics are isolated and cannot effectively characterize the whole of computational neuroscience research. Some general words, such as “conclusion” and “figure,” are also included in topics because a large amount of noise in full-text corpora affects topic recognition based on word distribution.

Different from LDA, the proposed AT-NeuroEAE can extract a group of events with rich semantics for outlining the whole research process:

• Brain mechanisms: The article reveals that “anxiety” activates the brain areas “cingulate gyrus” and “fusiform gyrus.”

• Experiment process: The experiments adopted the “mind reading task” with “chemosensory stimulus.” Researchers used “T1 weighted” “fMRI” device to collect data from “the donors.”

• Analytical process: The study got the “contrast map” by the “SPM5.” “dmn” was also obtained as the analytical result.

Because the extraction is based on pre-defined event categories, those general words can be effectively filtered.

**FIGURE 8:**
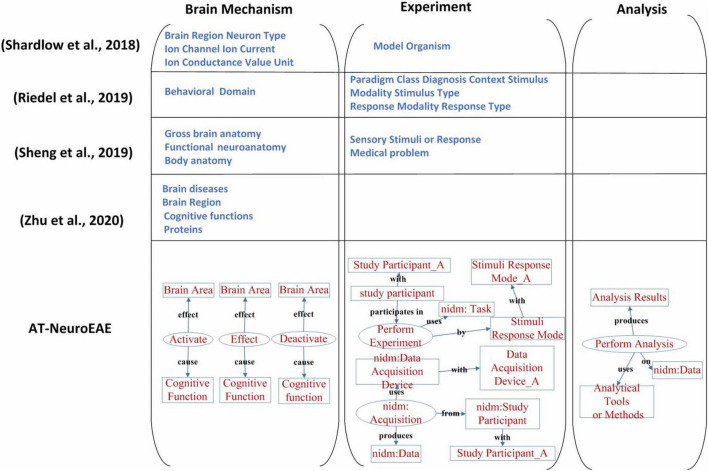
A comparison of neuroimaging entity/label categories/span of interest. The study on Shardlow et al. (2018) mainly focused on brain mechanism, especially multi-level brain structures. The study on Riedel et al. (2019) only took account of the experimental process. The study on Sheng et al. (2019) focused on brain mechanism and two experimental factors, including sensory stimuli or response and study participants’ medical problems. The study on Zhu et al. (2020) paid attention on pathology and mechanism of brain diseases. Our study is involved with the whole research process and extracted information is organized by events with rich semantics.

Named entity recognition is the current research focus in neuroimaging text mining. Because related studies (Shardlow et al., 2018; Riedel et al., 2019; Sheng et al., 2019) have different entity or label categories, this study only qualitatively compares their entity/label categories with the proposed AT-NeuroEAE. The result is shown in Figure 8.

The study on (Shardlow et al., 2018) mainly focused on brain mechanism, especially multi-level brain structures. The cognitive function and the research process were neglected. In contrast, the study on (Riedel et al., 2019) only took account of the experimental process and neglected brain mechanisms and the analytical process. The study of Sheng et al. (2019) focused on brain mechanism, but neglected the analysis process which is very important to understand and evaluate the conclusion about brain mechanism. About the experiment, only sensory stimuli or response and study participants’ medical problems were extracted. Some key experimental factors, including experimental tasks, data acquisition equipment, etc., have not been paid enough attention. The study of Zhu et al. (2020) paid attention on pathology and mechanism of brain diseases, and its spans of interests are limited to brain diseases, brain regions, cognitive functions, and proteins.

In this study, neuroimaging events related to brain mechanism and the whole research process were extracted. In the aspect of brain mechanism, three event categories “Activate,” “Deactivate,” and “Effect” represent not only location information but also physiological characteristics information such as brain network. In the aspect of experiment, the event category “Perform Experiment” represents as much information as the study on (Riedel et al., 2019) because the representations about stimuli, response, and tasks in neuroimaging articles are flexible and it is not necessary to distinguish the form and type of stimulus, task paradigm, and context in detail. The event category “Acquisition” represents the imaging devices and scanning parameters. In the aspect of analysis, the event category “Perform Analysis” represents three important factors and their relationships during neuroimaging data analysis, including methods/tools, results and brain areas. All extracted information in this study is not an isolated entity, but organized as a systematic information network in the form of events, which can better describe the whole research process.

## Conclusion

This study proposed an approach based on event extraction to realize research sharing-oriented neuroimaging provenance construction. Guided by the provenance model, the neuroimaging research process and result are modeled as six categories of neuroimaging event-containing attributes. A joint extraction model based on deep adversarial learning, called AT-NeuroEAE, is proposed to extract the defined neuroimaging events in a few-slot learning scenario. The experimental results on the PLOS ONE data set show that the model can realize the large-scale and low-cost neuroimaging provenance construction for open and FAIR research sharing in computational neuroscience.

## Software Dependencies

As described above, the analyses presented in this work rely on the following dependencies: numpy (van der Walt et al., 2011), pandas (McKinney, 2011), statsmodels (Seabold and Perktold, 2010), SciPy (Jones et al., 2001), scikit-learn (Pedregosa et al., 2011), IPython (Pérez and Granger, 2007), nltk (Bird et al., 2009), pdfminer (Shinyama, 2007), seaborn (Waskom et al., 2017), and many core libraries provided with Python 2.7.11. Additionally, the ontological expansion of Cognitive Atlas term weights was influenced by Poldrack et al. (2011).

## Data Availability Statement

The original contributions presented in the study are included in the article/Supplementary Material, further inquiries can be directed to the corresponding author/s.

## Author Contributions

SL, JC, and ZX developed the study design and performed the coding and analyses. All authors contributed to the implementation and contributed to the manuscript revision, and read and approved the submitted version.

## Conflict of Interest

The authors declare that the research was conducted in the absence of any commercial or financial relationships that could be construed as a potential conflict of interest.

## Publisher’s Note

All claims expressed in this article are solely those of the authors and do not necessarily represent those of their affiliated organizations, or those of the publisher, the editors and the reviewers. Any product that may be evaluated in this article, or claim that may be made by its manufacturer, is not guaranteed or endorsed by the publisher.

## Footnotes

1 It is difficult to distinguish information from knowledge clearly because they have varying definitions in different applications. In this study, “research information” refers to all descriptions about computational science in scientific literature. It is involved with not only research findings, such as coordinates of activated brain areas, but also the whole research process, from experimental design to data analysis.

2
http://www.brainmap.org/

3
http://www.neurosynth.org/

4
http://neurovault.org

5
http://www.w3.org/TR/prov-dm/

6
http://medical.nema.org/dicom/

7
https://github.com/incf-nidash/XCEDE

8
http://brainspell.org

9
https://github.com/wangmm88/BioNLP-ST-2016_BB3-event
